# Prognostic significance of RSPO1, WNT1, P16, WT1, and SDC1 expressions in invasive ductal carcinoma of the breast

**DOI:** 10.1186/1477-7819-11-314

**Published:** 2013-12-27

**Authors:** Eun Ji Choi, Jeong A Yun, Eun Kyoung Jeon, Hye Sung Won, Yoon Ho Ko, Su Young Kim

**Affiliations:** 1 Department of Pathology, The Catholic University of Korea, School of Medicine, Seochogu Banpodaero 222, Seoul 137-701, Republic of Korea; 2 Department of Internal Medicine, The Catholic University of Korea, School of Medicine, Seochogu Banpodaero 222, Seoul 137-701, Republic of Korea

**Keywords:** Breast, Carcinoma, Ductal, RSPO1 protein, SDC1 protein, WNT1 protein, WT1 protein

## Abstract

**Background:**

To better understand the mechanisms of the SDC1 expression in invasive ductal carcinoma, we studied the correlations between SDC1 expression and related gene expressions (RSPO1, WNT1, WT1, and P16).

**Methods:**

Using 100 cases of invasive ductal carcinoma tissue, we screened expressions of RSPO1, WNT1, WT1, P16, and SDC1 using immunohistochemistry. We analyzed the association between the immunoreactivities and clinicopathological parameters.

**Results:**

WT1 expression was associated with tumor grade. RSPO1 expression was associated with progesterone receptor expression. Expressions of RSPO1, WT1, and P16 were significantly associated with disease-free survival. RSPO1 and P16 showed statistically significant hazard ratios. SDC1 ectodomain expression was significantly associated only with P16 expression. Immunoreactivity of SDC1 cytoplasmic domain was associated with WT1 and WNT1. However, WNT1 expression failed to show a significant association with disease-free survival.

**Conclusions:**

RSPO1 and P16 immunoreactivity was found to be an independent prognostic indicator in invasive ductal cancer. Cytoplasmic expression of SDC1 is positively correlated with tumor-prone proteins (WT1 and WNT1) and membranous expression of SDC1 is positively correlated with the tumor suppressor (P16).

## Background

Carcinoma of the breast, besides skin cancer, is the most common malignancy in women and the number of women with breast cancer is increasing [1]. Among several histologic types of breast cancer, ductal carcinoma is generally referred to as adenocarcinoma without other designation and comprises the majority (79%) of breast cancer [1]. Patient’s age, age at menarche, and estrogen exposure are well known risk factors of breast cancer. There is currently a constant search for molecular markers to aid in the diagnosis of cancer and patient prognosis. To date, several genes and their products have been introduced to predict the prognosis of breast cancer patients, such as transmembrane protease serine 4 (TMPRSS4) [2], c-Kit [3], and syndecan-1 (SDC1) [4].

SDC1 is a member of the syndecan family, which is a group of heparan sulfate proteoglycans (HSPGs); four different types of syndecans are known in human [5]. The expression of HSPGs, including the core proteins and glycosaminoglycan chains, is altered in malignant tumors [6]. Among the syndecans, SDC1 is associated with various human cancers, including breast cancer. However, changes of SDC1 expression in tumors are not straightforward. Generally, SDC1 expression is reduced in most malignant tumors [7,8]. However, increased SDC1 expression has been reported in breast cancer [4] and pancreatic cancer [9]. Out of 13 HSPGs, only SDC1 has been shown as overexpressed in breast cancer [10]. In addition to quantitative changes in SDC1 expression, cleavage of SDC1 core protein in malignancy has been reported [6]. SDC1 consists of three domains; the ectodomain, the transmembrane domain, and the cytoplasmic domain [5]. Cleavage and shedding of the SDC1 ectodomain is found in multiple myeloma [11].

Clinical significance of SDC1 expression has been tested in several cancers. In gastric cancer, patients with SDC1-positive stroma had a worse outcome than patients with SDC1-negative stroma [12]. In colorectal cancer, SDC1 expression was associated with stage and grade of cancer [13]. Loss of epithelial SDC1 was associated with a more favorable prognosis in breast cancer [14] and SDC1 overexpression was associated with poor overall survival in estrogen receptor-negative patients [4]. Strong stromal staining, which is not found in normal breast tissue, was found in infiltrating ductal carcinomas [15].

However, the association of SDC1 overexpression with unfavorable prognosis is contradictory, with other reports claiming that SDC1 loss promotes invasion and metastasis and is associated with unfavorable outcome [7,11]. Because SDC1 shedding itself increases SDC1 expression in the cell [16], cytoplasmic expression of SDC1 may not represent the functional amount of SDC1. On the contrary, increased cytoplasmic expression may represent a lack of effective SDC1 on the cell surface and a reactive change by the feedback mechanism. Therefore, the cytoplasmic expression of SDC1 may mislead the clinical significance of the SDC1 product. To clarify this possible ambiguity in terms of clinical significance, a comparison of membranous and cytoplasmic expression of SDC1 is needed; this may resolve contradictory reports on the effects of SDC1 in tumor cells.

Although there are several reports showing an alteration of SDC1 in human cancers, none of them clearly explains the control mechanism or the effects of SDC1 expression. Considering that gene expression is affected by many other related gene expressions, evaluation of the gene expression associated with SDC1 may give us a clue to understand the regulation mechanism or affected pathways by SDC1 expression. According to previous reports, several genes have been associated with SDC1 expression. A strong correlation was reported between SDC1 expression and WT1 [17], and HSPGs have been shown to be necessary for the proper activity of WNT proteins [18]. Syndecans are also known to regulate WNT signaling. SDC1-null mice showed inhibition of mammary tumor development and WNT-dependent tumor initiation [19]. R-spondin proteins and P16 are closely related to the WNT/β-catenin pathway [20]. By evaluating these associated proteins, the alteration mechanism of SDC1 expression may be further elucidated.

In this study, we evaluated the clinical significance of SDC1 expression and that of the associated genes, including WT1, RSPO1, WNT1, and P16. Also, we compared several screening methods of SDC1 immunoreactivity to find the most informative one.

## Methods

### Patients and tumor samples

This study included tumor tissues surgically resected from 100 patients who visited Uijeongbu Mary’s Hospital and were diagnosed with invasive ductal carcinoma of the breast between 2002 and 2004. Patients’ age ranged between 29 and 77 (mean, 49.5) years old. All patients were diagnosed as invasive ductal carcinoma; 43 cases received adjuvant chemotherapy, 35 cases received hormone therapy, and 21 cases received adjuvant radiotherapy. Disease-free survival data (median, 62.8 months; range, 12.8 to 103.3 months) was available. The disease relapsed in 19 patients and 6 patients died of the disease. Using the tissues, tissue microarray blocks were constructed and used for immunohistochemical staining. Human tissue acquisition and its use followed the Institutional Review Board-approved protocol (CUMC11U058) from the Catholic University of Korea, School of Medicine.

### Immunohistochemistry

The immunohistochemical staining of breast cancer tissue followed the previously reported protocol [21]. Briefly, tissue sections were transferred to ProbeOn Plus slides (Fisher Scientific, Pittsburgh, PA, USA) and dried for 2 hours at 56°C in a drying oven (Agilent Technologies, Santa Clara, CA, USA). The sections were deparaffinized in xylene 3 times and rehydrated through 100%, 90%, 80%, and 70% ethanol and Tris-buffered saline (pH 7.4). For antigen retrieval, the tissues were immersed in 10 mM sodium citrate buffer (pH 6.0) and boiled in a microwave for 20 minutes. After treating the tissues with 3% hydrogen peroxide in phosphate buffered saline to quench endogenous peroxidase, the tissues were incubated with diluted primary antibody at 4°C overnight (Table 1). After incubating the tissue with biotinylated secondary antibody, the TSA HRP System (PerkinElmer, Waltham, MA, USA) was used to amplify signal intensity. For visualization, liquid DAB + substrate chromogen system (Dako, Glostrup, Denmark) was used. Immunoreactivity was classified according to the percentage of stained tumor cells; strong positive, >50% of cells stained; weak positive, 10% to 50% of cells stained; negative, <10% of cells stained. For SDC1, membranous staining and cytoplasmic staining was scored separately for comparison. For RSPO1, WNT1, and WT1, the cytoplasmic stain was counted. For P16, the nuclear stain was scored.

**Table 1:** The primary antibodies used in immunohistochemistry

**Target**	**Dilution factor**	**Host**	**Clone**	**Provider**
**SDC1, ectodomain**	1:40	Mouse monoclonal	B-A38	Abcam, Cambridge, UK
**SDC1, cytoplasmic domain**	1:100	Rabbit polyclonal		BioVision, Milpitas, USA
**P16**	1:10	Mouse monoclonal	JC8	Santa Cruz Biotechnology, Dallas, USA
**RSPO1**	1:500	Rabbit polyclonal		Abcam
**WNT1**	1:20	Rabbit polyclonal	H-89	Santa Cruz Biotechnology
**WT1**	1:500	Rabbit polyclonal	C-19	Santa Cruz Biotechnology

### Statistical analysis

Where appropriate, *χ*^2^ test or Fisher’s exact test were used to evaluate association of immunoreactivity with clinicopathologic features. For survival analysis, the Kaplan-Meier method and the non-parametric log-rank test were used. We used Cox’s multivariate proportional hazard model to determine hazard ratios of selected clinicopathologic parameters. We used R ver. 2.15 (R foundation, Vienna, Austria) for statistical tests.

## Results

### Patient characteristics

Most of the cases studied consisted of grades II (52 cases) and III (44 cases). The most frequent T stage was T2 (63 cases). There were 27 and 10 cases for T1 and T3 stages, respectively. Nodal stage was N0 and N1 in 42 and 36 cases, respectively. Estrogen receptor was positive in 62 cases and progesterone receptor was positive in 67 cases (Table 2).

**Table 2:** Patient pathological parameters

**Pathological parameters**		**Number of cases (total 100 cases)**
**Age**	<50	58
	≥50	42
**Grade**	I	4
	II	52
	III	44
**T stage**	T1	27
	T2	63
	T3	10
**N stage**	N0	42
	N1	36
	N2	17
	N3	5
**Estrogen receptor**	Negative	29
	Positive	62
	NA*	9
**Progesterone receptor**	Negative	24
	Positive	67
	NA*	9

*NA: Not available.

### SDC1 immunoreactivity

**Figure 1:**
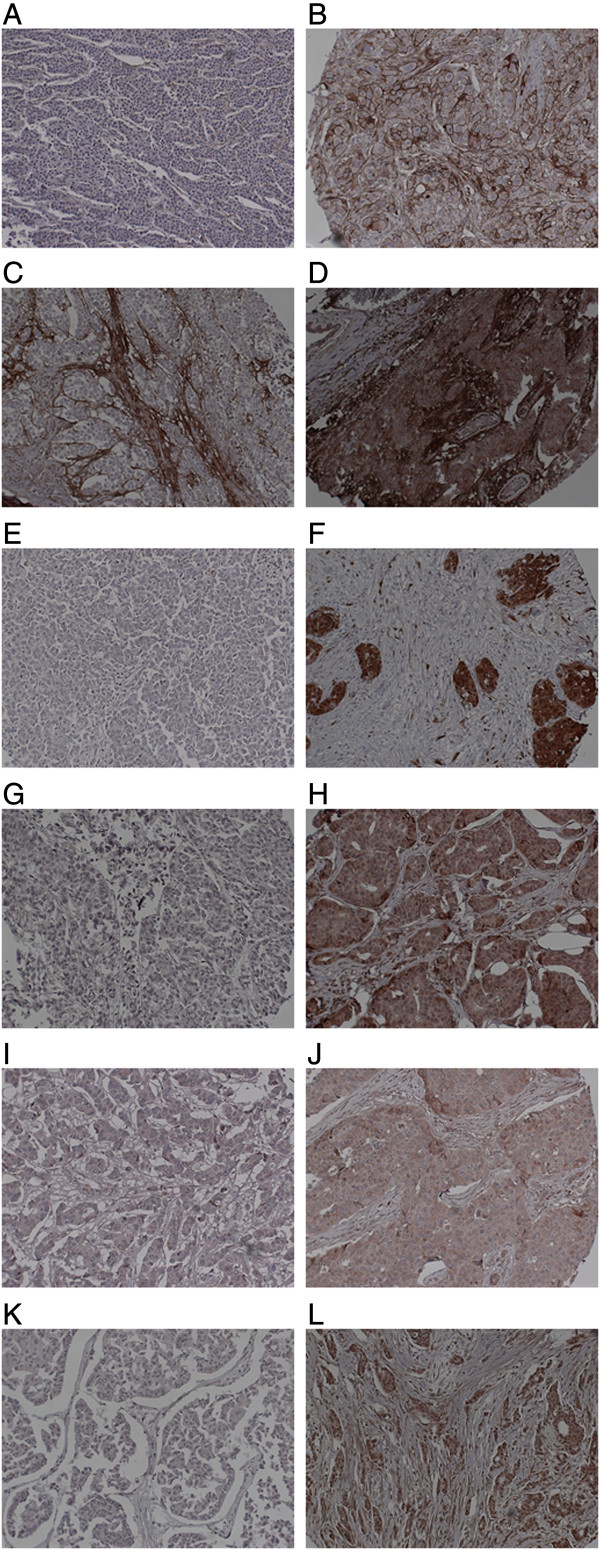
**Representative SDC1, P16, RSPO1, WNT1, and WT1 expression in invasive ductal carcinoma of the breast by immunohistochemistry. (A)** Negative for ectodomain of SDC1. **(B)** Positive for ectodomain of SDC1. **(C)** Negative for cytoplasmic domain of SDC1. **(D)** Positive for cytoplasmic domain of SDC1. **(E)** Negative for P16. **(F)** Positive for P16. **(G)** Negative for RSPO1. **(H)** Positive for RSPO1. **(I)** Negative for WNT1. **(J)** Positive for WNT1. **(K)** Negative for WT1. **(L)** Positive for WT1.

We used two different antibodies to detect different domains of SDC1 in the tumor cells. Anti-SDC1e antibody is directed against the ectodomain and anti-SDC1c antibody is against the cytoplasmic domain of the protein. For anti-SDC1e, a membranous stain was found in 29 cases and a cytoplasmic stain was found in 64 cases; 30 cases were negative for anti-SDC1e. For anti-SDC1c, a membranous stain was found in 33 cases and a cytoplasmic stain was found in 34 cases; 59 cases were negative for anti-SDC1c (Figure 1). Estrogen receptor was significantly associated with the membranous stain of anti-SDC1e (Table 3).

**Table 3:** WNT1, WT1, RSPO1, P16, and SDC1 expression in invasive ductal carcinoma in relation to clinicopathological parameters (n = number of cases)

	SDC1+ ** n	SDC1-** n	***P*** **value**	**WNT1+ n**	**WNT1- n**	***P*** **value**	**WT1+ n**	**WT1- n**	***P*** **value**	**RSPO1+ n**	**RSPO1- n**	***P*** **value**	**P16+ n**	**P16- n**	***P*** **value**
**T stage**			0.087			0.201			0.073			1.000			0.346
**1**	11	13		22	5		15	11		22	1		9	16	
**2–3**	18	55		48	25		24	45		64	5		17	55	
**N stage**			0.111			0.171			0.482			0.237			1.000
**0**	16	24		33	9		19	22		38	1		11	29	
**1–3**	13	44		37	21		20	34		48	5		15	42	
**Grade**			0.052			0.147			0.025*			0.694			0.911
**1–2**	21	33		43	13		28	26		48	4		14	41	
**3**	8	35		27	17		11	30		38	2		12	30	
**Estrogen**			0.035*			0.442			0.577			0.643			0.104
**receptor**				45	17		25	34		55	3		13	48	
**Positive**	14	47		18	11		9	18		24	2		11	16	
**Negative**	13	14													
**Progesterone**			0.124			0.276			0.545			0.019*			1.000
**receptor**				49	18		27	37		60	1		18	47	
**Positive**	23	42		14	10		7	15		19	4		6	17	
**Negative**	4	19													

*P <0.05.

**Membranous immunoreactivity of the antibody for SDC1 ectodomain.

### P16, RSPO1, WNT1, and WT1 expression

**Figure 2:**
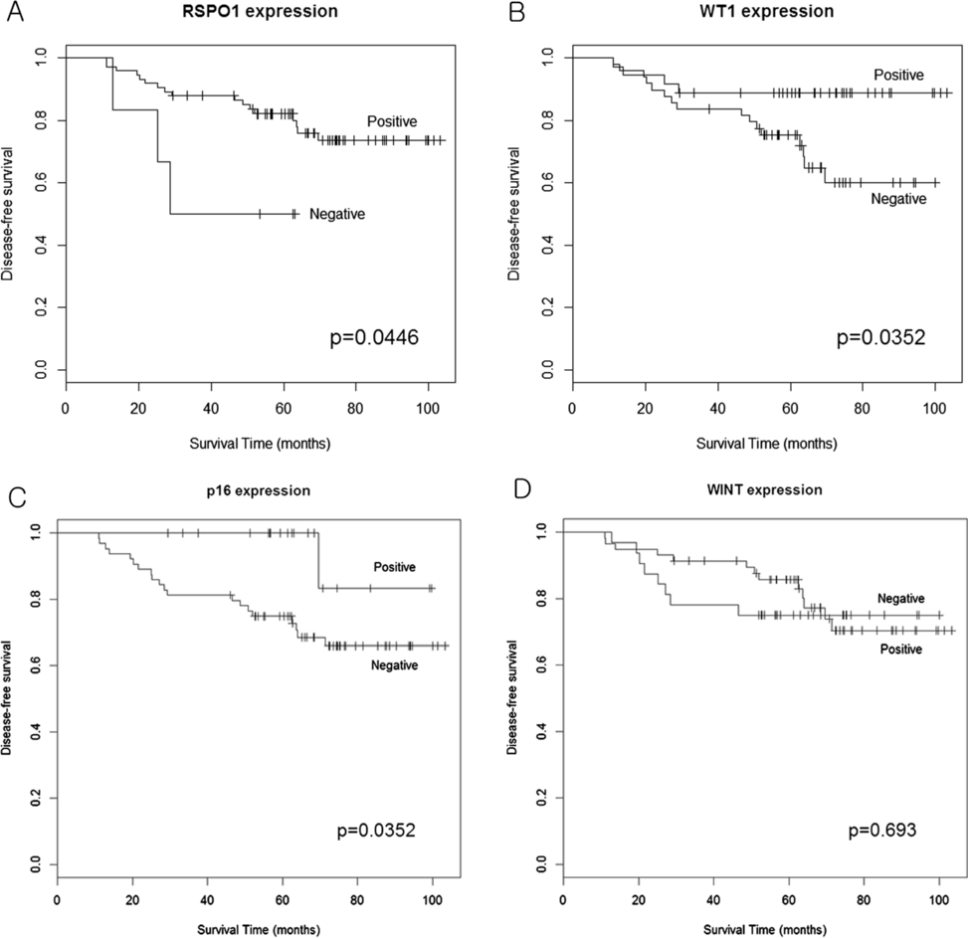
Survival analysis between groups showing different amount of expression of (A) RSPO1, (B) WT1, (C) P16, and (D) WNT1, respectively.

P16 was positive in 26 cases and RSPO1 was positive in 86 cases. WNT1 was positive in 70 cases and WT1 was positive in 39 cases (Figure 1). WT1 was associated with tumor grade (*P* = 0.025) and RSPO1 was associated with progesterone receptor expression (*P* = 0.019) (Table 3). P16 expression was significantly associated with membranous stain of anti-SDC1e (*P* = 0.008). The cytoplasmic stain of anti-SDC1c was associated with WT1 (*P* = 0.007) and WNT1 (*P* = 0.020) expressions (Table 4). RSPO1, WT1, and P16 positivity showed a significant disease-free survival difference between the positive group and the negative group. WNT1 expression failed to show a disease-free survival difference between the groups (Figure 2).

**Table 4:** Association between SDC1 scoring methods and P16, WT1, WNT1, and RSPO1 expressions

	**P16+ n**	**P16- n**	***P*** **value**	**WT1+ n**	**WT1- n**	***P*** **value**	**WNT1+ n**	**WNT1- n**	***P*** **value**	**RSPO1+ n**	**RSPO1- n**	***P*** **value**
**Membranous stain of SDC1e**			0.008**			0.863			0.944			0.405
**Positive**	11	10		7	13		15	5		33	1	
**Negative**	16	63		31	46		54	22		52	5	
**Cytoplasmic stain of SDC1e**			0.724			0.998			0.254			0.405
**Positive**	9	29		15	22		32	6		33	1	
**Negative**	18	44		23	37		41	16		52	5	
**Membranous stain of SDC1c**			0.684			0.250			1.000			1.000
**Positive**	3	5		1	7		6	2		8	0	
**Negative**	23	60		32	48		60	24		74	6	
**Cytoplasmic stain of SDC1c**			0.605			0.007**			0.020*			0.410
**Positive**	11	22		18	13		29	4		31	1	
**Negative**	15	43		15	42		37	22		51	5	

SDC1e: SDC1 ectodomain.

SDC1c: cytoplasmic domain of SDC1.

*P <0.05.

**P <0.01.

Multivariate analysis showed that RSPO1 and P16 expression were independent prognostic factors for disease-free survival (*P* = 0.029 and *P* = 0.027, respectively). On the contrary, SDC1 failed to show a significant hazard ratio (1.12, *P* = 0.841). Tumor grade, which is one of the well-known prognostic factors of breast cancer, showed a significant hazard ratio (4.48, *P* = 0.010) in our data (Table 5).

**Table 5:** Prognostic factors for disease-free survival selected by Cox’s multivariate proportional hazard regression model

	**Hazard ratio**	**95% confidence interval**	**Cox’s test *P *** **value**
**SDC1**	1.12	0.36–3.56	0.841
**Grade**	4.48	1.43–14.01	0.010**
**WNT1**	0.87	0.33–2.31	0.779
**WT1**	0.90	0.27–2.99	0.862
**RSPO1**	0.16	0.03–0.83	0.029*
**P16**	0.10	0.01–0.77	0.027*

*P <0.05, **P <0.01.

## Discussion

SDC1 expression is a major research topic for various human malignancies. Although immunohistochemistry using tissue microarray material is a general screening tool, the method of evaluating the immunoreactivity of SDC1 has not been thoroughly verified. As far as we know, all previous studies on SDC1 expression in tumor cells by immunohistochemistry do not differentiate the locations (the cytoplasmic membrane or the cytoplasm) of SDC1 expression. Because SDC1 is a transmembrane protein, elevated cytosolic expression may not directly relate to an increase in effective SDC1 protein. Ramani et al*.* reported that enzymatic degradation of the heparan sulfate chains increased SDC1 shedding and that enhanced SDC1shedding is accompanied by an increase in SDC1 expression [16]. Considering these findings, increased cytosolic expression of SDC1 may paradoxically represent a decrease in effective SDC1 protein on the cell surface or mask decreased expression of SDC1 on the cell surface when cytoplasmic and membranous expressions are considered to be equivalent. This necessitates differential scoring of SDC1 expression based on the location of expression in immunohistochemistry.

SDC1 in malignant cells may have two different forms; whole protein and remnant protein without ectodomain by shedding. The ectodomain is frequently cleaved from the SDC1 core protein in malignant cells. Removal of heparan sulfate from the cell surface accelerates SDC1 shedding [22]. To evaluate the clinical significance of the two different forms of SDC1, we used the antibodies for both the ectodomain and the cytoplasmic domain. We compared the two different scoring methods based on the locations of SDC1 expression to find the most informative way to evaluate SDC1 expression in tumor cells. As a result, we found that membranous staining by anti-SDC1e was associated with P16 expression. This association is clinically significant, since we showed the P16 expression is associated with disease-free survival. In addition, the cytoplasmic stain of anti-SDC1c was associated with WT1 and WNT1. Prognostic significance was found in WT1 expression, but not in WNT1 expression. These findings suggest that the ectodomain and the cytoplasmic domain of SDC1 may exert clinical effect through p16- or WT1-dependent pathways, respectively. Since the truncated form of SDC1, which is found in multiple myeloma, does not have an ectodomain, biological effects of SDC1 on malignant behavior of tumor cells may be dependent on the status of SDC1 size.

In the report claiming that loss of SDC1 was associated with a more favorable prognosis, both cytoplasmic and membranous patterns of SDC1 immunoreactivity were counted [4]. Because SDC1 shedding causes increased expression of SDC1 in the cytoplasm, immunoreactivity by the cytoplasm alone or with membranous immunoreactivity may show poor patient survival. Considering the molecular mechanism of SDC1, effective SDC1 scoring should be based on membranous staining patterns.

The mechanisms of how SDC1 expression is altered in tumor cells and the downstream pathways promoting invasion and metastasis are not clearly understood. One possible explanation can be found in the changes of HSulf-1 in tumor cells. Besides alteration of the HSPG core protein, alteration of heparan sulfate chains or sulfonation patterns were also noted in human cancers [23,24]. HSulfs, including HSulf-1, catalyze the desulfonation on trisulfated disaccharides [25]. Decreased expression of HSulf-1 stimulates cell growth by activation of the EGFR-ERK pathway [26].

In this study, we showed that the immunoreactivity of WT1, RSPO1, and P16 is significantly associated with a more favorable disease-free survival. Further, we also showed that RSPO1 and P16 are independent prognostic factors by Cox’s multivariate proportional hazard regression model. Tumor grade, which is a well-known prognostic factor, was the most significant prognostic factor in our model. P16 has also been introduced as a prognostic marker by other investigators [27] and we also confirmed the applicability in our data. In addition to the well-known factors, we introduced RSPO1 as a prognostic marker.

R-spondin proteins are secreted agonists of the canonical WNT/β-catenin signaling pathway [28]; there are four different types of R-spondin [20]. The relationship between R-spondin proteins and cancer was introduced in a study of RSPO1, whereby by regulating keratinocyte proliferation and differentiation, RSPO1 renders keratinocytes prone to squamous cell carcinoma [29]. In normal breast tissue of healthy women, RSPO1 was upregulated in the high serum estrogen level group and downregulated in the breast cancer group [30]. Our results add the prognostic significance of RSPO1 to its previous association with breast cancer [30]. Although R-spondins are expected to act through β-catenin stabilization and may synergize with WNT proteins [28], the survival analysis grouped by WNT1 expression suggests that RSPO1 exerts a protective effect through a pathway other than WNT1 signaling in invasive ductal cancer.

Manipulation of proteoglycan function is under testing for possible use to block cancer progression. A few glycosaminoglycan analogues, such as pentosan polysulfate and suramin, have entered clinical trials [24]. However, severe toxic effects prohibit the clinical use of these analogues. The detail understanding of altered control mechanisms of proteoglycans, such as SDC1, in tumor cells, will facilitate proteoglycan use in the diagnosis and treatment of cancer patients.

## Conclusions

In summary, we presented the association of SDC1 and related gene expressions by immunohistochemistry. The cellular compartment and target domain need to be mentioned in screening of SDC1 immunoreactivity. We confirmed the prognostic significance of P16 as previously suggested and newly introduced RSPO1 as a potential prognostic marker in invasive ductal cancer of the breast. Further, we showed P16 and WT1 expressions to be associated with the ectodomain and the cytoplasmic domain of SDC1 expression, respectively, with clinical significance. Because P16 and WT1 have opposite biological behaviors in tumorigenesis, screening of SDC1 expression by immunohistochemistry requires a clear description on expression location in the cell and the protein target domain.

## Abbreviations

HSPGs: Heparan sulfate proteoglycans; SDC1: Syndecan-1.

## Competing interests

The authors declare that they have no competing interests.

## Authors’ contributions

EJC carried out the immunohistochemistry and drafted the manuscript. JAY carried out the immunohistochemistry. EKJ, HKW, and YHK participated in collecting clinical samples and clinical data. SYK designed the study, analyzed the data, and finalized the manuscript. All authors read and approved the final manuscript.
